# Reduced peripheral CD4^+^ memory T-cell counts mark a mortality-associated immunophenotype in anti-MDA5 dermatomyositis

**DOI:** 10.3389/fimmu.2026.1924385

**Published:** 2026-08-12

**Authors:** Jinjing Qian, Ting Ma, Qiong Fu, Xinyue Lian, Sheng Chen

**Affiliations:** 1 Department of Rheumatology, Renji Hospital, School of Medicine, Shanghai Jiao Tong University, Shanghai, China; 2 Shanghai Immune Therapy Institute, Shanghai, China

**Keywords:** anti-MDA5 dermatomyositis, CD4^+^ memory T cells, lymphopenia, mortality, T-cell subsets

## Abstract

**Background:**

Anti-MDA5 dermatomyositis is often complicated by interstitial lung disease and early mortality. Peripheral lymphopenia has been linked to poor outcomes, but the routinely measured lymphocyte compartments underlying this signal remain incompletely defined. We used hierarchical flow-cytometric immunophenotyping to characterize the mortality-associated lymphopenic pattern.

**Methods:**

In this exploratory retrospective cohort, we studied 64 adults with anti-MDA5 dermatomyositis who had baseline lymphocyte subset testing and 180-day follow-up. We compared major lymphocyte populations and T-cell differentiation subsets between survivors and non-survivors. Associations with mortality were assessed using ROC, Kaplan-Meier, and Cox regression analyses.

**Results:**

Thirteen of 64 patients died by day 180. Non-survivors showed T-cell-dominant lymphopenia, most marked in CD4^+^ central and effector memory T cells. Median-split Kaplan-Meier analysis showed lower 180-day survival with lower CD4^+^ memory T-cell counts (log-rank P = 0.00058). Each 1-SD increase in CD4^+^ memory T-cell count was associated with lower mortality after age adjustment (aHR 0.16, 95% CI 0.04-0.63, P = 0.008). CD4^+^ memory T-cell count had an AUC of 0.80 (95% CI 0.68-0.91), comparable to total CD4^+^ T-cell count (AUC 0.78, 95% CI 0.65-0.90). Findings were similar after separate adjustment for LDH or KL-6 and among patients with baseline RP-ILD.

**Conclusions:**

Reduced peripheral CD4^+^ memory T-cell counts characterized a T-cell-dominant immunophenotype associated with 180-day mortality in anti-MDA5 dermatomyositis. These exploratory findings refine the established CD4^+^ lymphopenia signal and require validation in independent cohorts.

## Introduction

Anti-melanoma differentiation-associated gene 5 antibody-positive dermatomyositis (MDA5+DM) is a severe form of idiopathic inflammatory myopathy that is frequently complicated by rapidly progressive interstitial lung disease (RP-ILD) and early mortality (1–5). Although intensive immunosuppression and Janus kinase inhibitors have expanded treatment options (6, 7), short-term outcomes remain heterogeneous. The disease is therefore managed with close attention to inflammatory activity, pulmonary injury, and clinical deterioration. Routine immune-cell measurements may provide an additional way to characterize the biological state of patients at high risk.

Current risk assessment in MDA5+DM emphasizes systemic inflammation and lung injury, including serum ferritin, Krebs von den Lungen-6 (KL-6), high-resolution computed tomography findings, and composite models (3, 4, 8–11). Peripheral lymphopenia has also been associated with adverse outcomes (12–14). Immune-profiling studies have identified circulating signatures associated with clinical outcomes (15, 16), and single-cell profiling has revealed distinct adaptive immune hallmarks in MDA5+DM (17). A recent 268-patient study reported broad reductions across lymphocyte populations and identified immunophenotypic clusters with different RP-ILD and survival outcomes (18). These observations establish lymphopenia as a clinically relevant feature, but they do not determine whether the adverse signal reflects pan-lymphocyte loss, a predominantly T-cell pattern, or a narrower differentiation compartment within the CD4^+^ T-cell pool.

This distinction is biologically relevant because severe MDA5+DM combines interferon pathway activation, hyperferritinemia, and macrophage activation syndrome-like hyperinflammation (2, 10, 19–21). In parallel, tissue-focused studies suggest that pulmonary immune activation and monocyte-macrophage recruitment may coexist with changes in the peripheral immune compartment (17, 22). These processes could influence lymphocyte survival, trafficking, and differentiation. Routine flow cytometry cannot define the underlying mechanism, but it can identify the circulating cell populations that account for the observed lymphopenic phenotype.

CD4^+^ memory T cells arise following antigen exposure and support rapid secondary immune responses (23). Effector-memory CD4^+^ T cells can also interact with innate and myeloid inflammatory pathways (24, 25). The transcriptional and functional states of CD4^+^ T cells vary across diseases, which supports the value of disease-specific immunophenotypic resolution rather than reliance on broad cell counts alone (26). We therefore applied a hierarchical peripheral-blood flow-cytometry strategy. We first examined major lymphocyte compartments, then resolved the T-cell compartment into differentiation subsets, and finally assessed the association between the mortality-associated CD4^+^ memory compartment and 180-day mortality.

## Materials and methods

### Study design and patients

From August 2023 to March 2025, 74 adults with anti-MDA5 antibody-positive dermatomyositis met the clinical eligibility criteria and were evaluated at the Department of Rheumatology, Renji Hospital, Shanghai Jiao Tong University School of Medicine. Ten lacked baseline CD4^+^ memory subset measurements. The final analytic cohort included 64 patients with baseline lymphocyte-subset testing and 180-day follow-up data (**Supplementary Figure 1**). All fulfilled the modified Sontheimer criteria (27). Patients with overlap syndromes involving other systemic autoimmune diseases, coexisting malignancy, or active serious infection at admission were excluded.

The index blood sample was the first peripheral lymphocyte-subset test obtained within 24 hours of admission or evaluation at our center. Follow-up for 180-day mortality began on that date. Clinical characteristics, laboratory findings, treatment exposure, pulmonary involvement, respiratory support, intensive care admission, and causes of death were abstracted retrospectively from medical records.

Interstitial lung disease (ILD) was defined as interstitial abnormalities on chest high-resolution computed tomography (HRCT). RP-ILD was defined as worsening dyspnea and/or hypoxemia accompanied by radiological progression of ILD on HRCT within 3 months after respiratory symptom onset. RP-ILD status was determined from clinical and radiological information available at the index assessment. Pulmonary involvement was classified as no ILD, non-RP-ILD, or RP-ILD. Disease duration, PaO_2_/FiO_2_, and quantitative anti-MDA5 antibody values were recorded at baseline.

### Autoantibody testing and flow cytometry

Anti-MDA5 antibody positivity was confirmed at diagnosis using the EUROLINE Autoimmune Inflammatory Myopathies 16 Ag line-blot assay (Euroimmun, Lübeck, Germany), according to the manufacturer’s instructions. Quantitative anti-MDA5 antibody levels were summarized descriptively from enzyme-linked immunosorbent assay measurements in relative units per milliliter (RU/mL).

Peripheral lymphocyte subsets were measured by routine clinical whole-blood flow cytometry. Lymphocytes were identified by forward- and side-scatter characteristics and CD45 expression, followed by CD3^+^ T cells. Major populations included CD3^+^ T cells, CD19^+^ B cells, and CD3^-^CD16^+^ and/or CD56^+^ natural killer cells. Within the reported CD3^+^CD4^+^ and CD3^+^CD8^+^ compartments, differentiation subsets were defined by CD45RA and CCR7 expression using established categories (28, 29): naive (CD45RA^+^CCR7^+^), central memory (CD45RA^-^CCR7^+^), effector memory (CD45RA^-^CCR7^-^), and terminal effector memory cells re-expressing CD45RA (TEMRA; CD45RA^+^CCR7^-^). Double-positive and double-negative T cells were recorded separately and were not included in memory calculations. CD4^+^ memory count was the sum of absolute central memory and effector memory counts.

### Public single-cell analysis

Public sample-level source data from the published single-cell study HRA003082 were reanalyzed using the original cell-type annotations (17). Annotated CD4 central memory-like T cells (Tcm) and CD4 interferon-stimulated gene-positive cells (CD4 ISG) were examined separately. Active MDA5+DM samples were compared with a combined healthy-donor and control idiopathic inflammatory myopathy comparator using two-sided Wilcoxon rank-sum tests. Paired remission samples were displayed descriptively. This analysis used cell proportions, did not construct a flow-cytometry-equivalent pooled memory population, and was considered biological context rather than external validation.

### Outcome

The primary outcome was 180-day all-cause mortality, calculated from the date of the index lymphocyte-subset assessment.

### Statistical analysis

Continuous and categorical variables were compared using Student’s t-test, Wilcoxon rank-sum test, chi-square test, or Fisher’s exact test, as appropriate. Comparisons across T-cell differentiation subsets and biomarker correlations were adjusted using the Benjamini-Hochberg false discovery rate. Other baseline comparisons were descriptive. Associations between CD4^+^ memory T-cell count and conventional biomarkers were assessed using Spearman correlation. CD4^+^ memory T-cell count was analyzed per 1-standard-deviation (SD) increase in Cox proportional-hazards models. A cohort median split was used only to display Kaplan-Meier survival curves.

Discrimination for 180-day mortality was assessed using receiver operating characteristic (ROC) area under the curve (AUC) values with 95% confidence intervals. AUCs for CD4^+^ memory and total CD4^+^ T-cell counts were compared using paired DeLong testing and 5,000 patient level bootstrap resamples. Separate one covariate Cox models and an analysis restricted to patients with baseline RP-ILD were conducted as sensitivity analyses. Proportional-hazards assumptions were assessed using scaled Schoenfeld residuals.

Exploratory clinical-model comparisons before and after the addition of CD4^+^ memory T-cell count evaluated Harrell’s C, 180-day AUC, likelihood-ratio testing, calibration slope, Brier score, and decision-curve analysis. Full specifications and results are provided in the **Supplementary Methods** and **Supplementary Table 4**. Analyses were conducted in R using two-sided tests, with P<0.05 considered statistically significant.

## Results

### Clinical and pulmonary characteristics

Of the 64 patients, 51 survived and 13 died within 180 days. Non-survivors were older and had higher C-reactive protein (CRP), ferritin, KL-6, and lactate dehydrogenase (LDH) levels than survivors (**Table 1**). Pulmonary involvement at the index lymphocyte-subset assessment also differed between groups. All 13 non-survivors fulfilled RP-ILD criteria, compared with 32 of 51 survivors, and PaO_2_/FiO_2_ was lower in non-survivors than in survivors (median 118.90 versus 297.60, P<0.001). Twelve of the 13 deaths were attributed to respiratory failure or RP-ILD, and one was attributed to macrophage activation syndrome. Non-survivors more frequently required intensive care and higher-level respiratory support during the subsequent clinical course (**Table 1**).

**Table 1:** Baseline and clinical-course characteristics of survivors and non-survivors with MDA5+DM.

Clinical variable	Survivors (n=51)	Non-survivors (n=13)	P value
Baseline characteristics at index sampling			
Age, years	51.20 ± 12.07	62.85 ± 8.83	0.002
Female sex, n (%)	31 (60.8)	5 (38.5)	0.256
Skin ulcer, n (%)	6 (11.8)	0 (0.0)	0.333
Gottron rash, n (%)	33 (64.7)	5 (38.5)	0.160
Dysphagia, n (%)	1 (2.0)	1 (7.7)	0.368
Arthralgia, n (%)	18 (35.3)	2 (15.4)	0.201
Photosensitive rash, n (%)	9 (17.6)	1 (7.7)	0.672
White blood cell count, ×10^9^/L	6.66 (5.58, 8.94)	7.05 (6.09, 9.24)	0.489
Absolute neutrophil count, ×10^9^/L	5.53 (4.42, 8.02)	5.77 (5.09, 8.16)	0.297
Absolute lymphocyte count, ×10^9^/L	0.85 (0.61, 1.27)	0.59 (0.49, 0.84)	0.068
Neutrophil-to-lymphocyte ratio	6.59 (4.20, 13.38)	11.27 (6.66, 15.69)	0.133
C-reactive protein, mg/L	1.15 (0.25, 6.13)	6.34 (2.05, 15.44)	0.010
Erythrocyte sedimentation rate, mm/h	25.00 (17.00, 47.50)	29.00 (17.00, 39.00)	0.874
Ferritin, ng/mL	1206.40 (444.30, 1550.00)	1678.00 (1071.40, 2881.70)	0.016
KL-6, U/mL	996.00 (681.50, 1175.00)	2090.00 (1576.00, 3813.00)	<0.001
ALT, U/L	53.00 (32.50, 97.50)	47.00 (26.00, 54.00)	0.309
AST, U/L	51.00 (35.00, 85.50)	46.00 (29.00, 78.00)	0.701
CK, U/L	49.50 (33.00, 71.50)	62.00 (26.00, 96.00)	0.881
LDH, U/L	298.00 (243.50, 395.76)	511.00 (402.53, 1129.00)	<0.001
IFN-α, pg/mL	3.17 (2.60, 3.62)	3.17 (2.64, 4.85)	0.367
IFN-γ, pg/mL	2.98 (2.48, 3.68)	2.60 (2.43, 2.79)	0.045
Pre-admission prednisone-equivalent dose, mg/day	50.00 (40.00, 80.00)	60.00 (30.00, 100.00)	0.369
Pre-admission Janus kinase (JAK) inhibitor exposure, n (%)	17 (33.3)	3 (23.1)	0.739
Disease duration from symptom onset to admission, days	60.00 (30.00, 145.00)	90.00 (60.00, 120.00)	0.258
Pulmonary involvement pattern, n (%)			0.022
No ILD	3 (5.9)	0 (0.0)	
Non-RP-ILD	16 (31.4)	0 (0.0)	
RP-ILD	32 (62.7)	13 (100.0)	
Smoking history, n (%)	4 (7.8)	1 (7.7)	1.000
PaO_2_/FiO_2_	297.60 (242.05, 408.00)	118.90 (54.11, 184.80)	<0.001
Anti-MDA5 antibody level, RU/mL	128.77 (119.22, 140.64)	113.47 (92.36, 132.94)	0.134
Subsequent clinical course and outcomes			
Intensive care unit (ICU) admission, n (%)	8 (15.7)	10 (76.9)	<0.001
Highest respiratory support, n (%)			<0.001
No oxygen support	13 (25.5)	0 (0.0)	
Low-flow oxygen only	36 (70.6)	4 (30.8)	
High-flow nasal cannula (HFNC) without invasive ventilation	1 (2.0)	2 (15.4)	
Invasive mechanical ventilation	1 (2.0)	7 (53.8)	
JAK inhibitor treatment, n (%)	50 (98.0)	13 (100.0)	1.000
Calcineurin inhibitor treatment, n (%)	3 (5.9)	0 (0.0)	1.000
Intravenous immunoglobulin (IVIG) treatment, n (%)	10 (19.6)	3 (23.1)	0.717
Cause of death: respiratory failure/RP-ILD, n (%)	NA	12 (92.3)	NA
Cause of death: macrophage activation syndrome, n (%)	NA	1 (7.7)	NA

Data are presented as mean ± SD, median (IQR), or n (%). RP-ILD status was established at or before the index assessment. Pulmonary involvement and highest respiratory support were summarized as mutually exclusive categories; subsequent treatment categories were not mutually exclusive. P values are descriptive and were not adjusted for multiple comparisons. ALT, alanine aminotransferase; AST, aspartate aminotransferase; CK, creatine kinase; CRP, C-reactive protein; HFNC, high-flow nasal cannula; ICU, intensive care unit; IFN, interferon; ILD, interstitial lung disease; IVIG, intravenous immunoglobulin; JAK, Janus kinase; KL-6, Krebs von den Lungen-6; LDH, lactate dehydrogenase; NA, not applicable; PaO_2_/FiO_2_, arterial oxygen partial pressure/fraction of inspired oxygen ratio; RP-ILD, rapidly progressive interstitial lung disease.

### T-cell-dominant lymphopenic pattern

**Figure 1:**
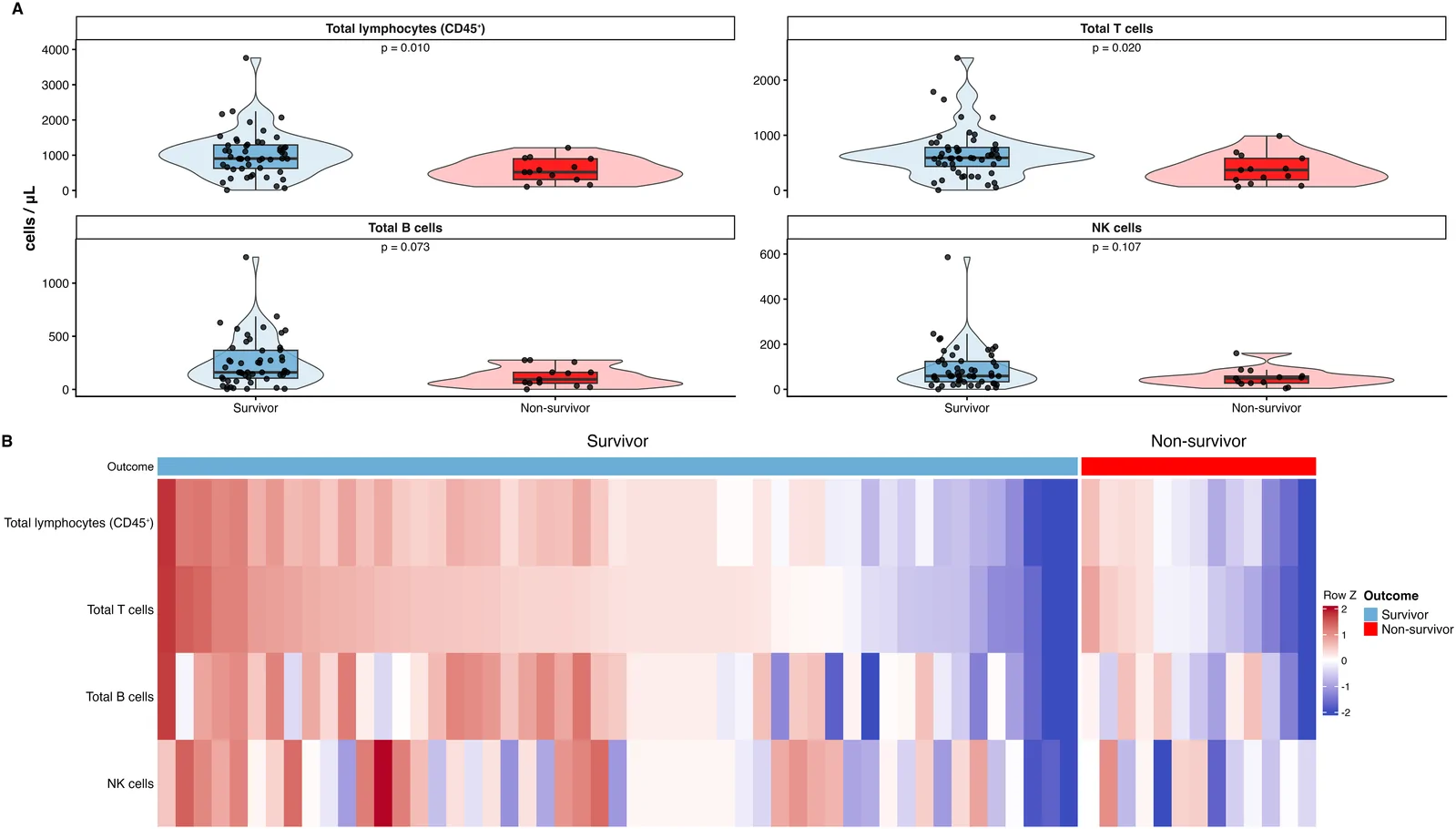
T-cell-dominant reduction among major lymphocyte subsets in MDA5+DM. **(A)** Absolute counts of total lymphocytes (CD45^+^), total T cells, total B cells and NK cells in survivors and non-survivors. FDR values are from two-sided Wilcoxon rank-sum tests with Benjamini-Hochberg adjustment across the four major-compartment comparisons. **(B)** Heatmap of row-wise z-score-normalized major immune-cell counts across patients, ordered by total T-cell count and annotated by outcome.

Routine blood counts are summarized in **Table 1**. Absolute lymphocyte count was numerically lower in non-survivors than in survivors (0.59 versus 0.85 ×10^9^/L, P = 0.068), whereas white blood cell count, absolute neutrophil count, and the neutrophil-to-lymphocyte ratio did not differ significantly. Among the major lymphocyte populations, total lymphocyte and total T-cell counts were lower in non-survivors after false discovery rate adjustment (both FDR = 0.040). The corresponding differences for total B cells and natural killer cells were not statistically significant (FDR = 0.097 and 0.107, respectively; **Figure 1**). These results indicated that the clearest between-group difference among major lymphocyte populations occurred in the T-cell compartment.

**Figure 2:**
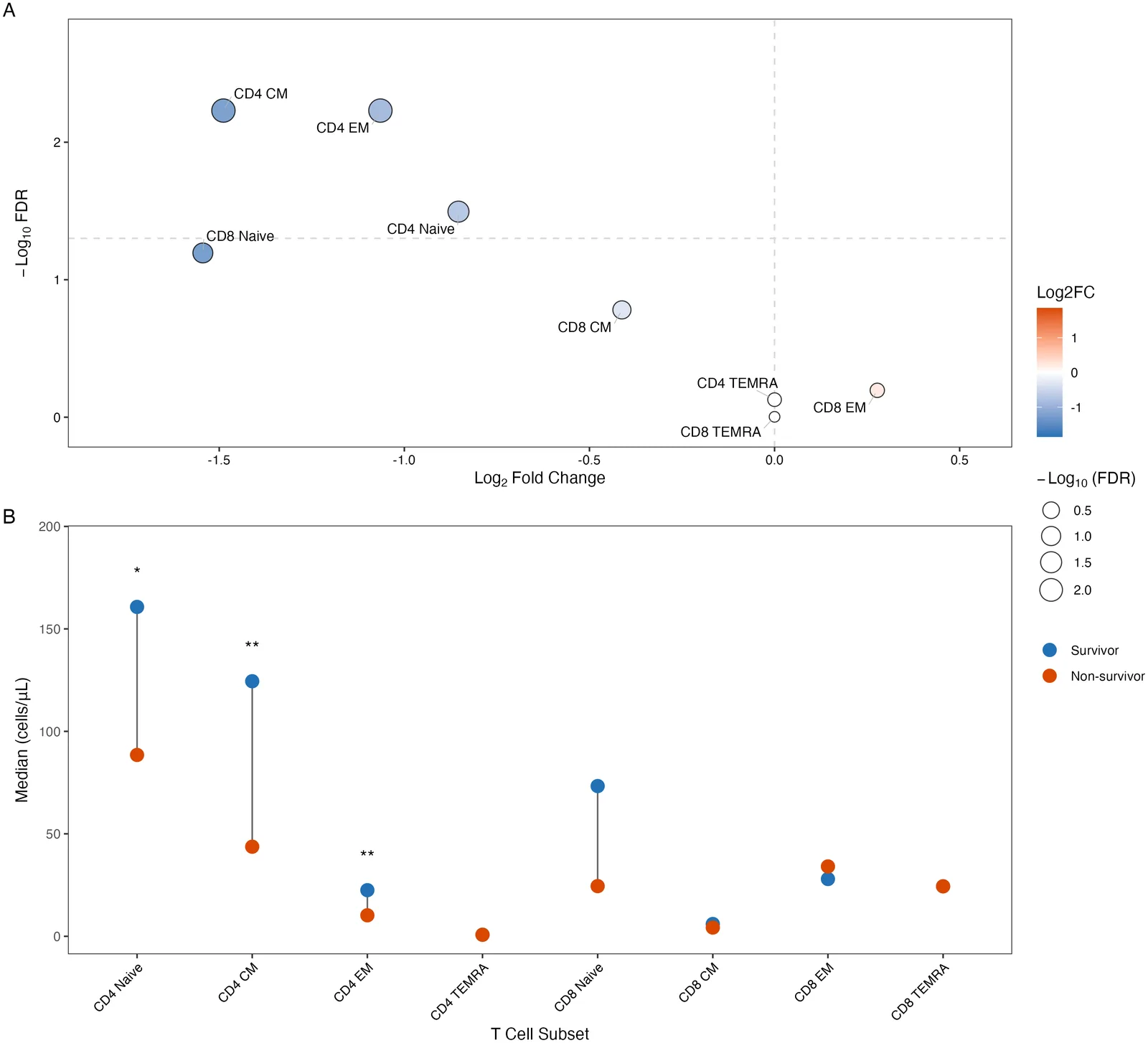
Peripheral T-cell subset differences between survivors and non-survivors. **(A)** Differential abundance plot for eight T-cell differentiation subsets. Log2 fold change was calculated as the non-survivor median relative to the survivor median. Negative values indicate lower counts in non-survivors. Point color indicates log2 fold change, and point size indicates -log10(FDR). The horizontal dashed line marks FDR = 0.05. **(B)** Median absolute counts of the same subsets in survivors and non-survivors. Lines connect group medians. Asterisks denote Benjamini-Hochberg-adjusted FDR thresholds: one asterisk for FDR<0.05 and two asterisks for FDR<0.01. CM, central memory; EM, effector memory; TEMRA, terminally differentiated effector memory cells re-expressing CD45RA.

Within the differentiation analysis, CD4^+^ naive, central memory, and effector memory counts were lower in non-survivors. Median central memory counts were 43.71 cells/μL in non-survivors and 124.47 cells/μL in survivors, and median effector memory counts were 10.24 and 22.51 cells/μL, respectively. Both central memory and effector memory comparisons remained significant after false discovery rate adjustment (FDR = 0.006 for each), whereas the remaining CD4^+^ and CD8^+^ differentiation subsets did not show comparably robust differences (**Figure 2**). Because the central memory and effector memory populations together represent the conventional circulating CD4^+^ memory compartment, their absolute counts were combined for subsequent mortality analyses. Results for CD4^+^ naive cells and related CD4^+^ compartment measures are provided in **Supplementary Table 5**.

### Associations between CD4^+^ memory T-cell count and conventional biomarkers

CD4^+^ memory T-cell count correlated inversely with LDH (rho=-0.40, raw P = 0.001, FDR = 0.004) and CRP (rho=-0.30, raw P = 0.017, FDR = 0.040). Correlations with ferritin, KL-6, IFN-α, and IFN-γ were weak and did not reach statistical significance after false discovery rate adjustment (**Supplementary Table 3**).

### Lower CD4^+^ memory T-cell counts were associated with 180-day mortality

**Figure 3:**
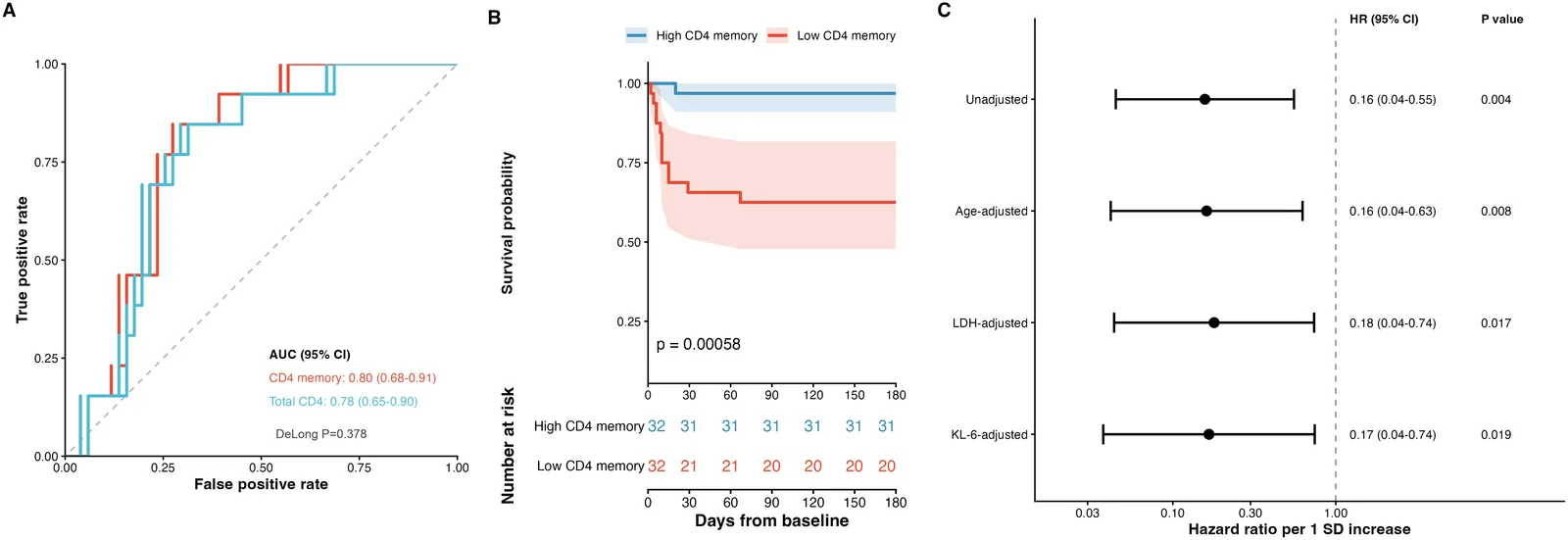
Association between peripheral CD4^+^ memory T-cell count and 180-day mortality. **(A)** ROC curves for CD4^+^ memory and total CD4^+^ T-cell counts; the paired DeLong P value was 0.378. **(B)** Kaplan-Meier survival curves with 95% confidence bands and numbers at risk for patients with baseline CD4^+^ memory T-cell counts below or above the cohort median. **(C)** Cox proportional-hazards estimates with 95% confidence intervals and P values per 1-SD increase in CD4^+^ memory T-cell count. Each row represents a separate Cox model.

Kaplan-Meier analysis showed lower 180-day survival in the CD4^+^ memory-low group than in the CD4^+^ memory-high group (log-rank P = 0.00058; **Figure 3B**). In continuous analysis, each 1-SD increase in CD4^+^ memory T-cell count was associated with lower mortality in the unadjusted model (hazard ratio [HR] 0.16, 95% confidence interval [CI] 0.04-0.55, P = 0.004) and after adjustment for age (adjusted HR 0.16, 95% CI 0.04-0.63, P = 0.008; **Figure 3C**).

CD4^+^ memory T-cell count had an AUC of 0.80 (95% CI 0.68-0.91) for 180-day mortality, compared with 0.78 (95% CI 0.65-0.90) for total CD4^+^ T-cell count. The paired DeLong comparison was not statistically significant (P = 0.378; **Figure 3A**). Patient-level bootstrap resampling yielded median AUCs of 0.80 for CD4^+^ memory and 0.78 for total CD4^+^ T cells, supporting a similar degree of within-cohort discrimination (**Supplementary Table 2**).

The association between CD4^+^ memory T-cell count and mortality was similar after separate adjustment for LDH, ferritin, KL-6, pre-admission prednisone-equivalent dose, pre-admission JAK inhibitor exposure, or other treatment variables (**Supplementary Table 1**). In the analysis restricted to 45 patients with baseline RP-ILD, lower CD4^+^ memory T-cell counts remained associated with mortality in the unadjusted model (HR 0.34, 95% CI 0.14-0.80, P = 0.014) and after age adjustment (HR 0.34, 95% CI 0.13-0.87, P = 0.024). Exploratory clinical-model comparisons showed modest numerical changes in apparent discrimination after adding CD4^+^ memory T-cell count, whereas calibration and decision-curve net benefit varied across models and threshold probabilities (**Supplementary Table 4**).

### Public single-cell analysis

Public source-data analysis showed lower proportions of annotated CD4 Tcm cells and higher proportions of CD4 ISG cells in active MDA5+DM than in the combined comparator group (P = 0.042 and P = 0.0010, respectively; **Supplementary Figure 2**). These sample-level observations describe altered CD4 T-cell states in active disease.

## Discussion

This exploratory retrospective study identified a T-cell-dominant lymphopenic immunophenotype in anti-MDA5 dermatomyositis. The hierarchical analysis first showed that the major between-group reduction was concentrated in the total T-cell compartment and then localized the most prominent differentiation-subset reductions to the CD4^+^ central memory and effector memory populations. The combined CD4^+^ memory count was associated with 180-day mortality in survival and continuous Cox analyses. This framework extends the broad observation of lymphopenia by specifying which routinely measured circulating T-cell compartment accounted for the strongest mortality-associated signal in this cohort.

Previous studies have linked lower total lymphocyte and CD4^+^ T-cell counts to poor outcomes in MDA5+DM, and more recent immunophenotypic work has described heterogeneous immune endotypes in this disease (12–18). Our findings are consistent with that literature while adding a differentiation-level view of the CD4^+^ compartment. The pattern was not one of uniform loss across all measured lymphocyte populations. Rather, total T-cell reduction accompanied prominent decreases in two antigen-experienced CD4^+^ populations. The accompanying decrease in CD4^+^ naive T cells suggests broader contraction of the CD4^+^ T-cell compartment rather than an isolated memory abnormality. However, exploratory analyses showed a less consistent mortality association for CD4^+^ naive T-cell count than for the combined CD4^+^ memory count, supporting CD4^+^ memory as the main readout at the compartment level. This observation does not establish CD4^+^ memory count as a superior prognostic marker to total CD4^+^ count. Its value is to provide a clinically accessible cellular context for the broader CD4^+^ lymphopenic phenotype.

CD4^+^ memory T cells preserve the capacity for rapid adaptive responses after antigen exposure and are shaped by sustained inflammatory environments (23). Effector-memory CD4^+^ cells can also influence innate and myeloid inflammatory programs (24, 25). In the present cohort, the inverse associations with LDH and CRP linked lower CD4^+^ memory counts to tissue injury and systemic inflammation, although the absence of clear correlations with ferritin, KL-6, and interferon measures suggests that these readouts capture partly distinct biological processes. The public single-cell analysis provided compatible, but non-equivalent, context by showing a lower annotated CD4 Tcm proportion and higher CD4 ISG proportion in active MDA5+DM. Because flow cytometry and single-cell annotation use different definitions and measurement scales, these results should not be interpreted as direct replication. Similar work in other chronic inflammatory settings also points to altered memory T-cell states during persistent immune activation (30).

Several mechanisms could contribute to reduced circulating CD4^+^ memory counts. Persistent interferon and RIG-I pathway activation may promote lymphocyte dysfunction or loss, while chemokine-mediated trafficking could redistribute activated T cells to inflamed tissues (19, 21, 31, 32). Pulmonary immune activation and monocyte-macrophage recruitment may further shape the peripheral immune landscape in severe MDA5+DM-related ILD (17, 22). Our single time point peripheral measurements cannot distinguish among these mechanisms. Longitudinal blood and tissue studies will be needed to determine whether the CD4^+^ memory pattern reflects altered survival, impaired maintenance, functional exhaustion, or tissue redistribution.

From a clinical perspective, CD4^+^ memory T-cell counts can be obtained from routine flow cytometry and may complement established measures of inflammation and lung injury. Their AUC was comparable to that of total CD4^+^ T-cell count, which supports refinement rather than replacement of the established CD4^+^ lymphopenia signal. The persistence of the association within patients who had already fulfilled RP-ILD criteria at baseline suggests immune heterogeneity even within severe pulmonary disease. Immune-state measures are also being explored as translational biomarkers in other disease contexts (33). The exploratory clinical-model comparisons were directionally compatible with complementary information, but they were not designed to establish a clinically actionable prediction rule. Independent prospective validation is required before CD4^+^ memory counts can be incorporated into risk assessment.

Several limitations should be considered. This was a single-center retrospective cohort restricted to patients with clinically available differentiation-subset testing, and only 13 deaths occurred. Pre-admission immunomodulatory treatment and baseline pulmonary severity may have confounded the observed associations. The analyses of discrimination and clinical-model performance were derived from the same cohort and require external validation. Finally, one peripheral-blood measurement cannot define the mechanism of lymphocyte reduction.

## Conclusions

Reduced peripheral CD4^+^ memory T-cell counts characterized a T-cell-dominant immunophenotype associated with 180-day mortality in anti-MDA5 dermatomyositis. This routine-flow-cytometry readout refines the broader CD4^+^ lymphopenia signal and warrants prospective multicenter evaluation alongside established markers of pulmonary and systemic disease severity.

## Data Availability

The raw data supporting the conclusions of this article will be made available by the authors, without undue reservation.
