# Supplementary material for: Reduced peripheral CD4+ memory T-cell counts mark a mortality-associated immunophenotype in anti-MDA5 dermatomyositis
Source: Front Immunol. 2026 Aug 12;17:1924385. doi: 10.3389/fimmu.2026.1924385 (PMC13506844; doi:10.3389/fimmu.2026.1924385)
Supplement: Supplementary file 1 [file SupplementaryFile1.docx]

**Supplementary material**

**Supplementary Methods**

**Flow cytometry workflow**

Peripheral lymphocyte subsets were measured from whole blood in the hospital clinical laboratory on a BD FACSCanto flow cytometer (BD Biosciences). Major lymphocyte subsets were processed using a stain-lyse-no-wash workflow. The CD4/CD8 differentiation panel used individually combined BD antibodies to CD4, CD8, CD45RA and CCR7 and included washing before acquisition. Absolute counts and population assignments were taken from the routine clinical report; raw acquisition files were not reanalyzed for post hoc mutually exclusive gating.

**Cox sensitivity analyses**

CD4⁺ memory T-cell count was analyzed per 1 SD increase. Separate Cox models adjusted for one covariate at a time: age, LDH, KL-6, ferritin, pre-admission prednisone-equivalent dose, pre-admission JAK inhibitor exposure, other immunomodulatory agent exposure, calcineurin inhibitor exposure or cyclophosphamide exposure. Other immunomodulatory agent exposure was defined as any pre-admission calcineurin inhibitor, cyclophosphamide, methotrexate or hydroxychloroquine exposure. Unadjusted and RP-ILD restricted analyses were also fitted. Proportional hazards assumptions were assessed using scaled Schoenfeld residuals. Complete case sample sizes are reported in Supplementary Table S1.

**Predictive performance analyses**

Three clinical base models were specified: age + ferritin + KL-6; age + ferritin + LDH; and age + ferritin + KL-6 + LDH. Each base model was compared with the corresponding extended model after adding baseline CD4⁺ memory T-cell count per 1 SD increase. Apparent discrimination was summarized using Harrell's C and 180-day ROC AUC, and nested model fit was compared using likelihood ratio tests.

The apparent calibration slope was estimated by logistic recalibration of binary 180-day mortality on the logit of Cox-predicted 180-day mortality risk. Apparent prediction error was summarized using the 180-day Brier score. Decision curve net benefit was evaluated at threshold probabilities from 10% to 50% in 5% increments, using treat-all and treat-none as reference strategies. Complete performance results are reported in Supplementary Table S4. No optimism correction based on resampling was applied to these model performance comparisons; they are exploratory apparent same cohort estimates rather than external validation or established clinical utility.

Supplementary Figure S1

Cohort assembly for the retrospective single-center analytic cohort. The source clinical dataset included 74 adults with anti-MDA5 dermatomyositis who met the clinical eligibility criteria and were evaluated at Renji Hospital from August 2023 to March 2025. Ten patients without available baseline CD4⁺ memory subset measurements were not included, leaving 64 patients with baseline lymphocyte subset testing and 180-day follow-up data.

Supplementary Figure S2

Public single-cell source data reanalysis of annotated CD4 T-cell states in MDA5+DM. Source data from a published single-cell study were reanalyzed using the original cell type annotations to compare CD4 central memory-like T cells (Tcm) and CD4 interferon-stimulated gene-positive cells (CD4 ISG) within circulating CD4 T cells across healthy donors (n=4), control idiopathic inflammatory myopathy samples (n=5), remission MDA5+DM samples (n=3), and active MDA5+DM samples (n=7). The corresponding raw single-cell data are available from NGDC GSA-Human under accession HRA003082. At the sample level, active MDA5+DM showed a lower annotated CD4 Tcm proportion (P=0.042) and a higher CD4 ISG proportion (P=0.0010) than the combined healthy donor and control idiopathic inflammatory myopathy comparator in two-sided Wilcoxon rank-sum tests. The remission samples were paired follow-up samples from participants represented in the active group and were displayed descriptively. P values refer only to the active-versus-combined-comparator analysis. Annotated CD4 Tcm is not equivalent to the CD4⁺ central memory plus effector memory compartment defined by CD45RA and CCR7 in flow cytometry, and this analysis provides biological context rather than external validation.

Supplementary Table S1

Cox proportional hazards sensitivity models for 180-day mortality. Hazard ratios are reported per 1 SD increase in peripheral CD4⁺ memory T-cell count. Each row represents a separate parsimonious model.

| Model | HR (95% CI) | P value | N | Events |
| --- | --- | --- | --- | --- |
| Unadjusted | 0.16 (0.04-0.55) | 0.0040 | 64 | 13 |
| Age-adjusted | 0.16 (0.04-0.63) | 0.0083 | 64 | 13 |
| LDH-adjusted | 0.18 (0.04-0.74) | 0.0171 | 64 | 13 |
| Ferritin-adjusted | 0.16 (0.05-0.54) | 0.0031 | 64 | 13 |
| KL-6-adjusted | 0.17 (0.04-0.74) | 0.0188 | 64 | 13 |
| Prednisone dose adjusted | 0.12 (0.03-0.52) | 0.0045 | 64 | 13 |
| JAK inhibitor adjusted | 0.09 (0.02-0.40) | 0.0017 | 64 | 13 |
| Other agent adjusted | 0.16 (0.05-0.59) | 0.0055 | 64 | 13 |
| Calcineurin inhibitor adjusted | 0.15 (0.04-0.54) | 0.0036 | 64 | 13 |
| Cyclophosphamide adjusted | 0.15 (0.04-0.53) | 0.0031 | 64 | 13 |
| RP-ILD restricted, unadjusted | 0.34 (0.14-0.80) | 0.0141 | 45 | 13 |
| RP-ILD restricted, age-adjusted | 0.34 (0.13-0.87) | 0.0243 | 45 | 13 |

Supplementary Table S2

ROC analysis and bootstrap uncertainty assessment for 180-day mortality. Lower cell counts were oriented toward higher mortality risk. Across 5,000 bootstrap resamples at the patient level, the median AUC was 0.80 (95% percentile interval 0.67-0.90) for CD4⁺ memory T cells and 0.78 (0.64-0.89) for total CD4⁺ T cells.

| Marker | AUC (95% CI) | N | Events |
| --- | --- | --- | --- |
| KL-6 | 0.89 (0.78-1.00) | 64 | 13 |
| LDH | 0.86 (0.74-0.98) | 64 | 13 |
| CD4⁺ memory T cells | 0.80 (0.68-0.91) | 64 | 13 |
| Total CD4⁺ T cells | 0.78 (0.65-0.90) | 64 | 13 |
| Ferritin | 0.72 (0.55-0.89) | 64 | 13 |
| Total T cells | 0.71 (0.55-0.87) | 64 | 13 |
| Total CD8⁺ T cells | 0.63 (0.44-0.81) | 64 | 13 |

Internal comparison of CD4⁺ memory T cells and total CD4⁺ T cells

| Comparison | AUC difference | DeLong P | Bootstrap difference (95% CI) | Bootstrap P | Successful bootstraps |
| --- | --- | --- | --- | --- | --- |
| CD4⁺ memory T cells vs total CD4⁺ T cells | 0.018 | 0.378 | 0.018 (-0.029 to 0.062) | 0.409 | 5000 |

Supplementary Table S3

Spearman correlations between peripheral CD4⁺ memory T-cell count and selected variables. Raw P values and Benjamini-Hochberg false discovery-rate values are shown.

| Variable | Spearman rho | P value | FDR | N |
| --- | --- | --- | --- | --- |
| Total T cells | 0.84 | <0.001 | 0.004 | 64 |
| LDH | -0.40 | 0.001 | 0.004 | 64 |
| CRP | -0.30 | 0.017 | 0.040 | 64 |
| KL-6 | -0.17 | 0.171 | 0.299 | 64 |
| IFN-α | 0.03 | 0.791 | 0.961 | 64 |
| IFN-γ | 0.02 | 0.905 | 0.961 | 64 |
| Ferritin | -0.01 | 0.961 | 0.961 | 64 |

**Supplementary Table S4**

Exploratory apparent predictive performance analyses for 180-day mortality in the 64-patient analytic cohort. Apparent Harrell's C and apparent 180-day ROC AUC compare parsimonious clinical models with and without CD4⁺ memory T-cell count; LRT P values compare nested Cox models. The apparent calibration slope was estimated by logistic recalibration of the binary 180-day outcome on the logit of the Cox-predicted 180-day mortality risk, and apparent 180-day Brier score summarizes overall prediction error. Decision curve net benefit was summarized at evaluated threshold probabilities from 10% to 50% in 5% increments with treat-all and treat-none reference strategies. NB, net benefit.

| **Model set** | **Model** | **N** | **Events** | **Harrell C** | 180-d AUC | **ΔC** | **ΔAUC** | **LRT P** | Risk-cal. slope | 180-d Brier |
| --- | --- | --- | --- | --- | --- | --- | --- | --- | --- | --- |
| Age + ferritin + KL-6 | Clinical model | 64 | 13 | 0.91 (0.86-0.97) | 0.95 (0.89-1.00) |  |  |  | 2.493 | 0.072 |
| Age + ferritin + KL-6 | Clinical model + CD4 memory | 64 | 13 | 0.93 (0.89-0.98) | 0.97 (0.93-1.00) | +0.02 | +0.02 | 0.001 | 1.279 | 0.058 |
| Age + ferritin + LDH | Clinical model | 64 | 13 | 0.90 (0.83-0.98) | 0.93 (0.85-1.00) |  |  |  | 1.464 | 0.071 |
| Age + ferritin + LDH | Clinical model + CD4 memory | 64 | 13 | 0.93 (0.89-0.98) | 0.96 (0.92-1.00) | +0.03 | +0.03 | 0.007 | 1.324 | 0.061 |
| Age + ferritin + KL-6 + LDH | Clinical model | 64 | 13 | 0.92 (0.86-0.98) | 0.95 (0.88-1.00) |  |  |  | 1.988 | 0.060 |
| Age + ferritin + KL-6 + LDH | Clinical model + CD4 memory | 64 | 13 | 0.94 (0.90-0.99) | 0.97 (0.94-1.00) | +0.03 | +0.03 | 0.004 | 1.200 | 0.055 |

Decision curve net benefit at evaluated threshold probabilities. Delta net benefit was calculated as the model including CD4⁺ memory T-cell count minus the corresponding clinical model.

| **Model set** | **Threshold** | **Clinical model NB** | **Clinical + CD4 memory NB** | **Δ NB** | **Treat-all NB** | **Treat-none NB** |
| --- | --- | --- | --- | --- | --- | --- |
| Age + ferritin + KL-6 | 0.10 | 0.174 | 0.175 | +0.002 | 0.115 | 0.000 |
| Age + ferritin + KL-6 | 0.15 | 0.142 | 0.171 | +0.029 | 0.062 | 0.000 |
| Age + ferritin + KL-6 | 0.20 | 0.148 | 0.168 | +0.020 | 0.004 | 0.000 |
| Age + ferritin + KL-6 | 0.25 | 0.146 | 0.146 | +0.000 | -0.062 | 0.000 |
| Age + ferritin + KL-6 | 0.30 | 0.134 | 0.129 | -0.004 | -0.138 | 0.000 |
| Age + ferritin + KL-6 | 0.35 | 0.125 | 0.115 | -0.010 | -0.226 | 0.000 |
| Age + ferritin + KL-6 | 0.40 | 0.125 | 0.109 | -0.016 | -0.328 | 0.000 |
| Age + ferritin + KL-6 | 0.45 | 0.094 | 0.102 | +0.009 | -0.449 | 0.000 |
| Age + ferritin + KL-6 | 0.50 | 0.094 | 0.125 | +0.031 | -0.594 | 0.000 |
| Age + ferritin + LDH | 0.10 | 0.141 | 0.186 | +0.045 | 0.115 | 0.000 |
| Age + ferritin + LDH | 0.15 | 0.147 | 0.165 | +0.018 | 0.062 | 0.000 |
| Age + ferritin + LDH | 0.20 | 0.141 | 0.148 | +0.008 | 0.004 | 0.000 |
| Age + ferritin + LDH | 0.25 | 0.141 | 0.125 | -0.016 | -0.062 | 0.000 |
| Age + ferritin + LDH | 0.30 | 0.152 | 0.136 | -0.016 | -0.138 | 0.000 |
| Age + ferritin + LDH | 0.35 | 0.124 | 0.132 | +0.008 | -0.226 | 0.000 |
| Age + ferritin + LDH | 0.40 | 0.115 | 0.130 | +0.016 | -0.328 | 0.000 |
| Age + ferritin + LDH | 0.45 | 0.112 | 0.128 | +0.016 | -0.449 | 0.000 |
| Age + ferritin + LDH | 0.50 | 0.125 | 0.125 | +0.000 | -0.594 | 0.000 |
| Age + ferritin + KL-6 + LDH | 0.10 | 0.160 | 0.191 | +0.031 | 0.115 | 0.000 |
| Age + ferritin + KL-6 + LDH | 0.15 | 0.150 | 0.174 | +0.024 | 0.062 | 0.000 |
| Age + ferritin + KL-6 + LDH | 0.20 | 0.145 | 0.137 | -0.008 | 0.004 | 0.000 |
| Age + ferritin + KL-6 + LDH | 0.25 | 0.146 | 0.141 | -0.005 | -0.062 | 0.000 |
| Age + ferritin + KL-6 + LDH | 0.30 | 0.156 | 0.121 | -0.036 | -0.138 | 0.000 |
| Age + ferritin + KL-6 + LDH | 0.35 | 0.141 | 0.115 | -0.025 | -0.226 | 0.000 |
| Age + ferritin + KL-6 + LDH | 0.40 | 0.141 | 0.109 | -0.031 | -0.328 | 0.000 |
| Age + ferritin + KL-6 + LDH | 0.45 | 0.141 | 0.128 | -0.013 | -0.449 | 0.000 |
| Age + ferritin + KL-6 + LDH | 0.50 | 0.125 | 0.125 | +0.000 | -0.594 | 0.000 |

These predictive performance analyses were exploratory and describe model performance within the cohort. Differences were calculated from unrounded estimates and may not equal differences between the displayed rounded values.

Supplementary Table S5

Exploratory analyses of CD4⁺ naive T-cell counts and related CD4⁺ T-cell compartments. Values are cells/µL. Hazard ratios are reported per 1 SD increase in cell count. Lower marker values were oriented toward higher 180-day mortality for AUC analyses. P values are exploratory.

A. Descriptive comparisons and ROC analysis.

| **Marker** | **Survivors** | **Non-survivors** | **Wilcoxon P** | **AUC (95% CI)** |
| --- | --- | --- | --- | --- |
| CD4⁺ naive T cells | 160.73 (102.75-289.96) | 88.49 (41.97-132.59) | 0.012 | 0.73 (0.59-0.86) |
| CD4⁺ central memory T cells | 124.47 (63.80-183.57) | 43.71 (34.08-63.16) | 0.001 | 0.79 (0.67-0.90) |
| CD4⁺ effector memory T cells | 22.51 (16.82-48.09) | 10.24 (7.85-17.41) | 0.001 | 0.79 (0.67-0.92) |
| CD4⁺ memory T cells | 151.45 (77.04-223.00) | 64.97 (39.65-71.81) | 0.001 | 0.80 (0.68-0.91) |
| Total CD4⁺ T cells | 350.25 (196.47-518.84) | 147.84 (92.48-200.84) | 0.002 | 0.78 (0.65-0.90) |

B. Cox proportional hazards analyses.

| Marker | Unadjusted HR (95% CI) | P | Age-adjusted HR (95% CI) | P |
| --- | --- | --- | --- | --- |
| CD4⁺ naive T cells | 0.22 (0.06-0.82) | 0.025 | 0.27 (0.07-1.04) | 0.057 |
| CD4⁺ central memory T cells | 0.16 (0.05-0.59) | 0.006 | 0.16 (0.04-0.63) | 0.009 |
| CD4⁺ effector memory T cells | 0.16 (0.04-0.64) | 0.010 | 0.20 (0.05-0.82) | 0.025 |
| CD4⁺ memory T cells | 0.16 (0.04-0.55) | 0.004 | 0.16 (0.04-0.63) | 0.008 |
| Total CD4⁺ T cells | 0.20 (0.06-0.62) | 0.006 | 0.21 (0.06-0.72) | 0.013 |
